# Norcantharidin inhibits tumor growth and vasculogenic mimicry of human gallbladder carcinomas by suppression of the PI3-K/MMPs/Ln-5γ2 signaling pathway

**DOI:** 10.1186/1471-2407-14-193

**Published:** 2014-03-15

**Authors:** Jing-Tao Zhang, Wei Sun, Wen-Zhong Zhang, Chun-Yan Ge, Zhong-Yan Liu, Ze-Ming Zhao, Xing-Sui Lu, Yue-Zu Fan

**Affiliations:** 1 Department of Surgery, Tongji Hospital, Tongji University School of Medicine, Tongji University, Shanghai 200065, P.R. China; 2 Department of Surgery, Shanghai Tenth People’s Hospital, Tongji University School of Medicine, Tongji University, Shanghai 200072, P.R. China; 3 Department of Surgery, Shanghai Pudong New Area People’s Hospital, Shanghai 201299, P.R. China; 4 Department of Oncology, Shanghai Yangpu Geriatric Hospital, Shanghai 200090, P.R. China

**Keywords:** Gallbladder neoplasm, Norcantharidin, Vasculogenic mimicry, 3-dimensional matrix, Xenograft model, Signaling pathway

## Abstract

**Background:**

Vasculogenic mimicry (VM) is a novel tumor blood supply in some highly aggressive malignant tumors. Recently, we reported VM existed in gallbladder carcinomas (GBCs) and the formation of the special passage through the activation of the PI3K/MMPs/Ln-5γ2 signaling pathway. GBC is a highly aggressive malignant tumor with disappointing treatments and a poor prognosis. Norcantharidin (NCTD) has shown to have multiple antitumor activities against GBCs, etc; however the exact mechanism is not thoroughly elucidated. In this study, we firstly investigated the anti-VM activity of NCTD as a VM inhibitor for GBCs and its underlying mechanisms.

**Methods:**

*In vitro* and *in vivo* experiments to determine the effects of NCTD on proliferation, invasion, migration, VM formation, hemodynamic and tumor growth of GBC-SD cells and xenografts were respectively done by proliferation, invasion, migration assays, H&E staining and CD31-PAS double stainings, optic/electron microscopy, tumor assay, and dynamic micro-MRA. Further, immunohistochemistry, immunofluorescence, Western blotting and RT-PCR were respectively used to examine expression of VM signaling-related markers PI3-K, MMP-2, MT1-MMP and Ln-5γ2 in GBC-SD cells and xenografts *in vitro* and *in vivo*.

**Results:**

After treatment with NCTD, proliferation, invasion, migration of GBC-SD cells were inhibited; GBC-SD cells and xenografts were unable to form VM-like structures; tumor center-VM region of the xenografts exhibited a decreased signal in intensity; then cell or xenograft growth was inhibited. Whereas all of untreated GBC-SD cells and xenografts formed VM-like structures with the same conditions; the xenograft center-VM region exhibited a gradually increased signal; and facilitated cell or xenograft growth. Furthermore, expression of MMP-2 and MT1-MMP products from sections/supernates of 3-D matrices and the xenografts, and expression of PI3-K, MMP-2, MM1-MMP and Ln-5γ2 proteins/mRNAs of the xenografts were all decreased in NCTD or TIMP-2 group; (all *P* < 0.01, *vs*. control group); NCTD down-regulated expression of these VM signaling-related markers *in vitro* and *in vivo*.

**Conclusions:**

NCTD inhibited tumor growth and VM of human GBCs in vitro and in vivo by suppression of the PI3-K/MMPs/Ln-5γ2 signaling pathway. It is firstly concluded that NCTD may be a potential anti-VM agent for human GBCs.

## Background

Gallbladder carcinoma (GBC) is the most common biliary tract cancer (BTC), the fifth or sixth common malignant neoplasm of the digestive tract and the leading cause of cancer-related deaths in West countries and China [1-5]. It commonly presents at an advanced stage, and has limited therapeutic options such as low surgical resection rate, disappointing chemotherapy and radiotherapy; moreover, diagnostic delay, high local recurrence and distant metastasis, and biological behavior of the tumor, the prognosis is very poor [1,6-13]. Therefore, comprehension of the special biological behaviors and the molecular events in gallbladder carcinogenesis, and development of novel anticancer or molecularly targeted therapeutics in advanced GBC are very necessary, and remain challenging [12,13]. Recent developments in targeted therapeutics, directed against several key signalling pathways in BTC, including epidermal growth factor receptor, angiogenesis, and the mitogen-activated protein kinase pathway appear promising [13].

The growth and metastasis of the tumor depend on an effective microcirculation. The formation of a microcirculation can occur *via* the traditionally recognized mechanisms of vasculogenesis and angiogenesis and the recently found vasculogenic mimicry (VM). VM, a newly-defined pattern of tumor blood supply, provides a special passage without endothelial cells and conspicuously different from angiogenesis and vasculogenesis [14], describes the unique ability of highly aggressive tumor cells to express endothelial cell-associated genes and form extracellular matrix (ECM)-rich, patterned tubular networks when cultured on a three-dimensional (3-D) matrix, and is associated with a poor prognosis for the patients with some aggressive malignant tumors such as melanoma [14,15], breast cancer [16], hepatocellular carcinoma [17], gastric adenocarcinoma [18], and colorectal cancer [19], etc.. We previously reported that VM existed in human GBCs and GBCs by both 3-D matrices of highly aggressive GBC-SD cells *in vitro* and GBC-SD nude mouse xenografts *in vivo* and correlated with the patient’s poor prognosis [20-22]. We identified that the formation of VM in human GBCs through the activation of the phosphoinositide 3 ­kinase/matrix metalloproteinases/laminin 5γ2 (PI3K/MMPs/Ln-5γ2) signaling pathway in the 3-D matrices of GBC-SD cells *in vitro* and GBC-SD nude mouse xenografts *in vivo*[23,24]. Because differential endothelial cells involved in angiogenesis and VM, and their different molecular regulation mechanisms are key targets in cancer therapy, some experiments confirmed that simple application angiogenic inhibitors have no effect on VM [25]. So, it should be considered to develop new antivascular therapeutic agents that target both angiogenesis and VM, in especial, anti-VM therapy for tumor VM.

Evidence has shown that traditional Chinese medicines contain anticancer ingredient. Norcantharidin (NCTD) is a demethylated and low-cytotoxic derivative of cantharidin with anti-tumor properties, an active ingredient of the traditional Chinese medicine Mylabris; is currently synthesized from furan and maleic anhydride *via* the DielsAlder reaction [26-28]. It has been reported that NCTD inhibits the proliferation and growth of a variety of human tumor cells and is used in clinic to treat human cancers, e.g., hepatic, gastric, colorectal and ovarian carcinoma because of its effective anticancer activity, fewer side effects and leukocytosis [26-31]. We have reported that NCTD has multiple antitumor activities against GBCs *in vitro* and *in vivo*[32-34]. However, the exact mechanism responsible for the NCTD antitumor is not thoroughly elucidated. In this study, we further investigated the anti-VM activity of NCTD as a VM inhibitor for human GBCs and its underlying mechanisms. The results showed that NCTD inhibits tumor growth and VM of human GBCs by suppression of the PI3-K/MMPs/Ln-5γ2 signaling pathway *in vitro* and *in vivo*. Thus, we firstly concluded that NCTD may be a potential anti-VM agent for human GBCs.

## Methods

### Cell culture

Establishment of human gallbladder carcinoma GBC-SD cell lines have been described previously [22] and were maintained in Dulbecco’s modified Eagle’s media (DMEM, Gibco, USA) supplemented with 10% fetal bovine serum (FBS, Hangzhou Sijiqing Bioproducts, China) and 10^5^ U · ml^−1^ penicillin and streptomycin (Shanghai Pharmaceutical Works, China) in an incubator (Forma series II HEPA Class 100, Thermo, USA) at 37°C with 5% carbon dioxide (CO_2_).

### Proliferation assay *in vitro*

Cultured GBC-SD cell suspensions were used in acute toxicity test [32]. Maximal (100 μg · ml^−1^) or minimal (5 μg · ml^−1^) effective dose was calculated respectively from pro-experiment. Cells were grown in a 96-well plate (3 × 10^5^ cells/ml · 100 μl/well) in culture medium overnight, then treated with various concentrations of NCTD (injection solution: 5 mg · ml^−1^; Jiangsu Kangxi Pharmaceutical Works, China) in fresh culture medium at 37°C in 5% CO_2_ for 24 hr. The tetrazolium-based colorimetric assay (MTT; Sigma, MO, USA) was used to determine the effect of NCTD on proliferation of GBC-SD cells. The optical densities (*A* value) at 540 nm were measured with an enzyme-linked immunosorbent assay (ELISA) reader (Biorad model 450, Sigma, Germany). The *A*540 value of the experimental groups was divided by the *A*540 value of untreated controls and presented as a percentage of the cells. Inhibitory percent of NCTD on GBC-SD cells (%) = (1-*A*540 value in the experimental group ∕*A*540 value of control group) × 100%. Three separate experiments were carried out. The concentration of drug giving 50% growth inhibition (IC_50_) was calculated from the formula IC_50_ = lg^−1^[Xm-I (p-0.5)].

### Invasion assay *in vitro*

The 35-mm, 6-well Transwell membranes (Coster, USA) were used to assess the *in vitro* invasiveness of GBC-SD cells. Briefly, a polyester (PET) membrane with 8-μm pores was uniformity coated with a defined basement membrane matrix consisting of 50 μl Matrigel (Becton Dickinson, USA) mixture which diluted with serum-free DMEM (2 volumes *versus* 1 volume) over night at 4°C and used as the intervening barrier to invasion. Upper wells of the chamber were respectively filled with 1 ml serum-free DMEM containing 2 × 10^5.^ ml^−1^ GBC-SD cells (n = 3). Cells were untreated (control group) and treated with 100 nM tissue inhibitor of matrix metalloproteinase-2 (TIMP-2) recombinant protein (Sigma, Germany; TIMP_2_ group) or 28 μg · ml^−1^(1/2 IC_50_) of NCTD (NCTD group) in fresh culture medium (0.3 ml/every chamber). Lower wells of the chamber were filled with 3 ml serum-free DMEM containing 1 × MITO + (Collaborative Biomedical, Bedford, MA). After 24-hr in a humidified incubator at 37°C with 5% CO_2_, cells that had invaded through the basement membrane were stained with H&E, and counted by a light microscope. Invasiveness was calculated as the number of cells that had successfully invaded through the matrix-coated membrane to the lower wells. Briefly, quantification was done by calculating the number of cells in 5 independent microscopic fields at a 400-fold magnification. Experiments were performed in duplicate and repeated three times with consistent results.

### Collagen gel contraction i.e. migration assay *in vitro*

Collagen gel suspensions for GBC-SD cell lines are prepared by mixing 250 μl of a suspension that contained 3 × 10^6.^ml^−1^ into 250 μl of undiluted rat-tail collagen type I (Sigma, Germany; 4.25 mg^.^ml^−1^) dripped into sterilized 35-mm petridishes that contained 2 ml culture media to prevent adhesion of the collagen to the glass substrate. The suspensions are allowed to polymerize for 1 hr at room temperature before these culture dishes were placed in the 37°C with 5% CO_2_ incubator. Cells were untreated (control group) and treated with 100 nM TIMP-2 recombinant protein (Sigma, Germany; TIMP_2_ group) or 28 μg · ml^−1^(1/2 IC_50_) of NCTD (NCTD group) for 24 hrs. Gel contraction was defined as the relative change in the gel size, measured in two dimensions, including maximum and minimum diameters. Gel measurements were recorded daily, and the culture medium was changed every one day. Contraction index (CI) was calculated as follows: CI = 1-(D-D_0_)^2^ × 100%, where D is the primary diameter of rat-tail collagen type I, D_0_ is the average of maximum and minimum diameters of gel. All experiments were performed in triplicate.

### Network formation assay *in vitro*

Matrigel and rat-tail type I collagen 3-D matrices were prepared as described previously [22]. Cells were allowed to adhere to matrix, and untreated (control group) and treated with 100 nM TIMP-2 recombinant protein (Sigma, Germany; TIMP_2_ group) or 28 μg · ml^−1^ of NCTD (NCTD group) for 2 days. For experiments designed to analyze the ability of the cells to engage in VM using a phase contrast microscopy (Olympus IX70, Japan). The images were taken digitally using a Zeiss Televal inverted microscopy (Carl Zeiss, Inc., Thornwood, NY) and camera (Nickon, Japan) at the time indicated.

### Tumor xenograft assay *in vivo*

Balb/c nu/nu mice (equal numbers of male and female mice, 4-week old, about 20 g) were provided by Shanghai Laboratory Animal Center, Chinese Academy of Sciences and housed in specific pathogen free (SPF) condition. All of procedures were performed on nude mice according to the official recommendations of the Chinese Community Guidelines. Tumor xenograft assay of GBC-SD cells *in vivo* was performed as described previously [22,24,34]. The mice, by 2 weeks when a tumor xenograft was apparent in all mice axilback, were randomly divided into a control group (*n* = 6) receiving intraperitoneal (i.p.) injections of 0.1 ml normal saline alone twice each week, a NCTD group (*n* = 6, each mouse receiving i.p. injections of 28 mg · kg^−1^ NCTD at a dose of 1/5 LD_50_ given in 0.1 ml of normal saline, as described previously [34]), and a TIMP-2 recombinant protein (Sigma, Germany; *n* = 6, each mouse receiving intratumoral injection of 100 nM) group, twice each week for 6 weeks in all. Xenograft size i.e. the maximum diameter (a) and minimum diameter (b) was measured with calipers two times each week. The tumor volume was calculated by the following formula: V (cm^3^) =1/6π*ab*^2^. Also, tumor inhibitory rate of each group was respectively evaluated. Tumor inhibitory rate = (volume in the control group - volume in the experimental group)/volume in the control group × 100%.

### Immunohistochemistry *in vitro* and *in vivo*

Immunohistochemistry *in vitro* and *in vivo* included H&E staining, periodic acid-Schiff (PAS) staining, CD_31_-PAS double stainings, and the determination of matrix metalloproteinase-2 (MMP-2) or membrane type 1-MMP (MT1-MMP) protein for sections and supernates from the cell culture tissues and sections of GBS-SD nude mouse xenografts. H&E staining, PAS staining and CD_31_-PAS double stainings were performed as indicated previously [22]. MMP-2 and MT1-MMP proteins from sections of 3-D culture samples and GBC-SD xenografts were determined by streptavidin-biotin complex (SABC) method as described previously [24]. Primary antibody [MMP-2 (1:200), MT1-MMP (1:100); Rabbit polyclonal antibody], biotinylated secondary antibody, SABC reagents and 3, 3-diaminobenzidine (DAB) solution were from Wuhan Boster, China. Sections were observed under an optic microscope with × 10 and × 40 objectives (Olympus CH-2, Japan). For negative control, the slides were addressed in phosphate buffer solution (PBS) in place of primary antibody. Ten sample slides in each group were selected by analysis. More than 10 visual fields were observed or more than 500 cells counted per slide. In addition, MMP-2 and MT1-MMP proteins from supernates of 3-D culture samples were determined by ELISA as indicated previously [24]. The supernates from each group and the diluted standard solutions were added into 2 multiple wells, 2 zero adjusting wells, and a control tetramethylbenzidine (TMB) well. The former two wells were added in order with biotinylated antibody (MMP-2, Wuhan Boster; MT1-MMP, DR, USA), ABC reagents and TMB solution (Wuhan Boster), respectively; the control TMB well were didn’t added in order with MMP-2, MT1-MMP, ABC reagents. The optical densities at 450 nm were needed to be measured using an ELISA reader (Biorad model, Sigma, Germany).

### Electron microscopy *in vitro* and *in vivo*

For scanning electron microscopy (SEM) and transmission electron microscopy (TEM), 3-D culture samples of GBC-SD cells and fresh tissues of GBC-SD nude mouse xenografts (0.5 mm^3^) were fixed in cold 2.5% glutaraldehyde in 0.1 mol^.^L^−1^ of sodium cacodylate buffer and postfixed in a solution of 1% osmium tetroxide, dehydrated, and embedded in a standard fashion. The specimens were then either embedded, sectioned, and stained by routine means for a JEOL-1230 TEM, or critically point-dried, and sputter-coated with gold for a Hitachi S-520 SEM.

### Hemodynamic assay of the xenografts’ VM *in vivo*

Hemodynamic assay of GBC-SD nude mouse xenografts were examined by a dynamic micro-magnetic resonance angiography (micro-MRA; MRI is a 1.5 T superconductive magnet unit from Marconic, USA) as described previously [22]. The anesthetized xenograft nude mice (n = 3, 7 weeks old, 35 ± 3 grams) placed at the center of the coils were injected I.V. in the tail vein with human adult serum gadopentetic acid dimeglumine salt injection [HAS-Gd-DTPA, 0.50 mmol (Gd) · ml^−1^, Mr = 60-100kD, 0.1 mmol(Gd) · kg^−1^, Schering, Germany] before sacrifice. Micro-MRA was performed to analyze hemodynamic in the VM (central tumor) regions [22]. The images were acquired before injection of the contrast agents and 2, 5, and 15 minutes after injection. Three regions of interset (ROI) in the central area and the marginal area of the xenografts were observed and time-coursed pixel numbers per mm^3^ were counted. Two experiments were performed on these three gated ROI. All of the data were obtained directly from the MRA analyzer and were expressed as the mean ± SD.

### Indirect immunofluorescence detection *in vivo*

PI3-K, MMP-2, MT1-MMP and Ln-5 γ2 protein products from GBC-SD xenografts of each group were determined by indirect immunofluorescence method as described previously [24]. The frozen sections (4 μm) of the xenografts from each group were pretreated, added in order with 50 μl (1:100) primary antibody (PI3-K: mouse anti-human polyclonal antibody, Acris Antibodies GmbH, USA; MMP-2, MT1-MMP: rabbit polyclonal antibody, Wuhan Boster; Ln-5γ2: mouse anti-human polyclonal antibody, Santa Cruz), biotinylated secondary antibody (1:100; goat anti-rabbit IgG-FITC/GGHL-15 F, or goat anti-mouse IgG-FITC/GGHL-90 F, Immunology Consultants Laboratory, USA), respectively. Then, sections were rinsed in TBS solution and distilled water, mounted coverslip using buffer glycerine, and observed under a fluorescence microscope (Nikon, Japan). For negative control, the slides were treated with PBS in place of primary antibody. Ten sample slides in each group were chosen by analysis. More than 10 visual fields were observed per slide. Expression of each VM signal-related protein on slides of the xenografts showed a yellowgreen fluorescent dyeing. Fluorescent dyeing intensity was classed into -, ±, +, ++, +++, ++++. Of them, - ~ +: negative expression, ≥++: positive expression.

### Western blotting *in vivo*

PI3-K, MMP-2, MT1-MMP and Ln-5 γ2 proteins from GBC-SD xenografts of each group were determined by Western blot analysis as described previously [22]. Cells were lysed. The supernatant was recovered. BCA protein was determined with a protein quantitative kit (KangChen, KC-430, China). Then, an aliquot of 20 mg of proteins was subjected to sodium dodecyl sulfate-polyacrylamide gel electrophoresis (SDS- PAGE) for electrophoresis under reducing condition, and were then transferred to a PVDF membrane. An hour after being blocked with PBS containing 5% non-fat milk, the membrane was incubated overnight, was then added in order with each primary antibody [mouse anti-human antibody, 1:3000; PI3-K (P85-a): Acris Antibodies GmbH; MMP-2, MT1-MMP: Wuhan Boster; Ln-5γ2: Santa Cruz], and mouse anti-human GAPDH antibody (1:10000; Kangcheng Bioengineering, Shanghai) diluted with PBST containing 5% non-fat milk at 4°C, an appropriate anti-mouse or anti-rabbit HRP-labeled secondary antibody (1:5000; Kangcheng Bioengineering). The target proteins were visualized by an enhanced chemiluminescent (ECL) reagent (KC™ Chemiluminescent Kit, KangChen, KC-420, China), imaged on the Bio-Rad chemiluminescence imager. The gray value and gray coefficient ratio of every protein were analyzed and calculated with Image J analysis software.

### RT-PCR analysis *in vivo*

PI3-K, MMP-2, MM1-MMP and Ln-5γ2 mRNAs from GBC-SD xenografts of each group were respectively determined by reverse transcription-polymerase chain reaction (RT-PCR) assay. RT-PCR was performed as described by the manufacturer. Total RNA from the xenograft cells of each group was prepared using the Trizol reagent (Invitrogen, USA). Concentration of RNA was determined by the absorption at 260 ~ 280. PCR amplifications were performed with gene-specific primers (Table 1) with annealing temperature and number of amplification cycles optimized using cDNA from the xenograft cells in each group. PCR amplification reactions were performed as follows: 1 cycle of 94°C for 5 min; 35 cycle of 94°C for 10 ~ 22 sec, 57 ~ 60°C for 15 ~ 20 sec, 72°C for 20 sec, 82 ~ 86°C (fluorescence collection) for 5 ~ 10 sec; 1 cycle of 72 ~ 99°C for 5 min. GAPDH primers were used as control for PCR amplication. 10 μL PCR products were placed onto 15 g · L^−1^ agarose gel and observed by EB (Ethidium bromide, Huamei Bioengineering Company, China) staining using the ABI Prism 7300 SDS software.

**Table 1:** VM signaling-related markers

**Gene**	**PCR primers (forward-reverse)**	**Amplification size (bp)**	**Cycle no.**
PI3-K	5′-*TGTCGCAGCCCAGGTAGATT-3*′	269	35
	5′-*CAGGAGGTGGTCGGGTCAAG*3-′		
MMP-2	5′-*TCTGAGGGTTGGTGGGATTGG-3*′	290	35
	5′-*AAGAGCGTGAAGTTTGGAAGCA-*3′		
MM1-MMP	5′-*CAAAGGCAGAACAGCCAGAGG*3-′	180	35
	5′-*ACAGGGACCAACAGGAGCAAG-*3′		
Ln-5γ2	5′-*ACACGGGAGATTGCTACTCG*3-′	123	35
	5′-*ACCCATTGTGACAGGGACAT-*3′		
GAPDH	5′-*CCTCTATGCCAACACAGTGC-*3′	211	35 ~ 40
	5′*GTACTCCTGCTTGCTGATCC-*3′		

### Statistical analysis

All data were expressed as mean ± SD and performed using SAS (9.0 version software, SAS Institute Inc., Cary, NC, USA). Statistical analyses to determine significance were tested with the *χ*^2^, *F* or Student-Newman-Keuls *t* tests. *P* < 0.05 was considered statistically significant.

## Results

### NCTD inhibits proliferation of GBC-SD cells *in vitro*

**Figure 1:**
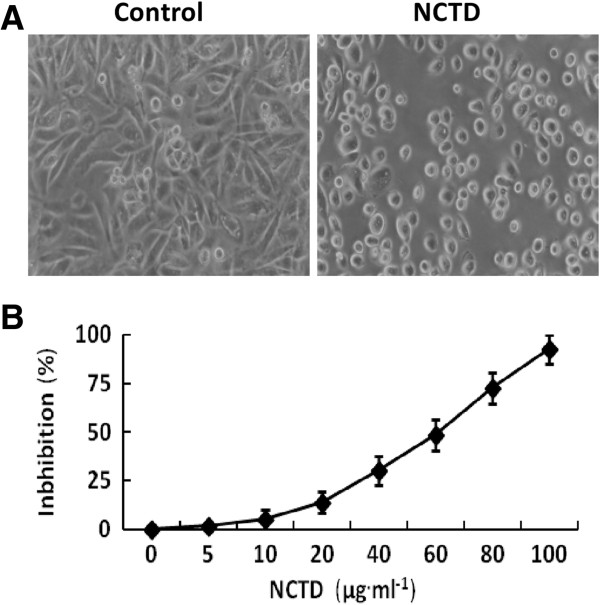
**Inhibitory effect of NCTD on proliferation of GBC-SD cells *in vitro*. (A)** Histomorphologic of GBC-SD cells under an inverted optic microscope (original magnification, ×200) at 24^th^ hr: predominantly shuttle-shape cells, with abundant cytoplasm and clear nuclei in control group; visible float or aggregation cells, with nuclear condensation and nuclear fragmentation in NCTD group (1/2 IC_50_ NCTD). **(B)** The dose-response curves of NCTD effect on GBC-SD cells with the IC_50_ value of 56.18 μg · ml^−1^.

MTT assay was used to determine the effect of NCTD on proliferation of GBC-SD cells. We found that the cultured GBC-SD cells began to growth at 6^th^ hr, maturated at 24^th^ hr, which were predominantly of shuttle-shape or accumulation, with abundant cytoplasm, clear nuclei in control group; after NCTD treatment, the morphology of GBC-SD cells showed visible cell aggregation, float, nuclear condensation or fragmentation, cataclysm, apoptotic bodies, or even death (Figure 1A). Furthermore, NCTD inhibited markedly proliferation of GBC-SD cells in a dose-dependent manner with the IC_50_ value 56.18 μg · ml^−1^ (Figure 1B).

### NCTD inhibits invasion of GBC-SD cells *in vitro*

**Figure 2:**
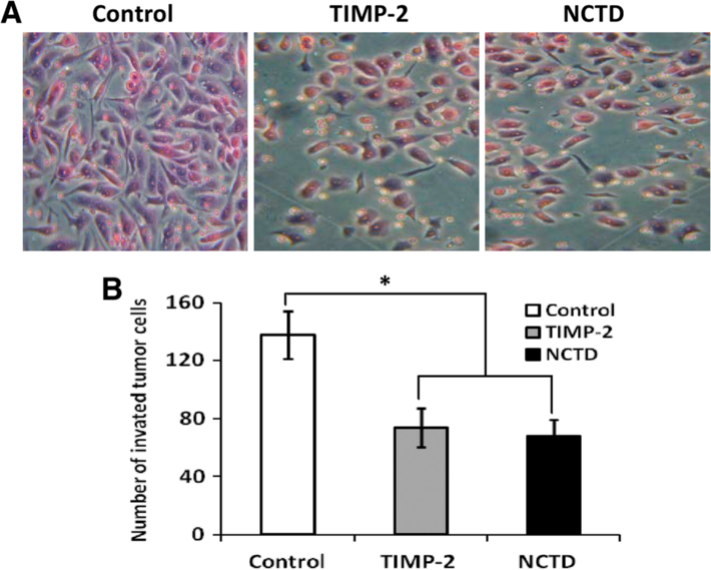
**Inhibitory effect of NCTD on invasion of GBC-SD cells *in vitro*. (A)** Representative histomorphologic of GBC-SD cells (original magnification, ×200) with H & E staining under an optic microscope. **(B)** The invaded number of GBC-SD cells in control group, TIMP-2 group and NCTD group. The invaded number of GBC-SD cells in TIMP-2 or NCTD group was much less than that of control group (*P* < 0.001), without different CI between TIMP-2 group and NCTD group.

The Transwell plates were used to measure the *in vitro* ability of GBC-SD cells to invade a basement membrane matrix. We found that GBC-SD cells in control group passed more of the Transwell membrane and had more invasive capability than TIMP-2 or NCTD group *in vitro* (Figure 2A); the number of passing membrane cells i.e, invated tumor cells in TIMP-2 or NCTD group markedly decreased (Figure 2B; *P* < 0.001). Thus, NCTD, similarly to TIMP-2, inhibited significantly invasion of GBC-SD cells *in vitro*.

### NCTD inhibits migration of GBC-SD cells *in vitro*

**Figure 3:**
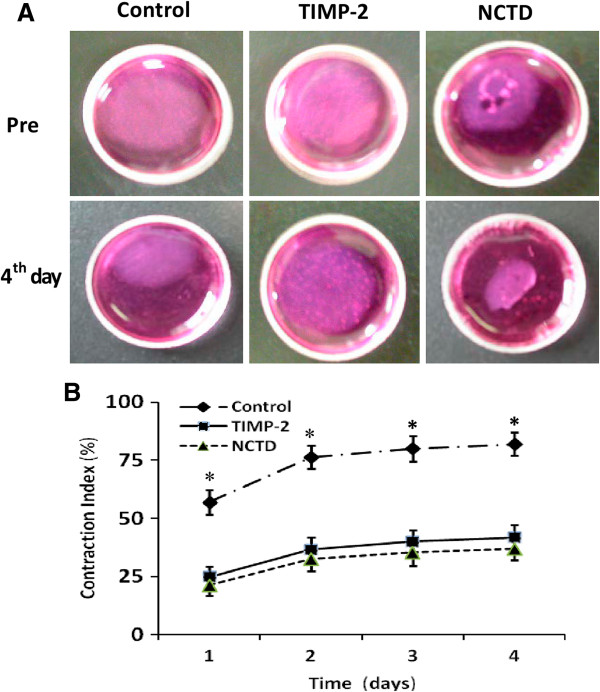
**Inhibitory effect of NCTD on migration of GBC-SD cells *in vitro*. (A)** Representative pictures of collagen gel contraction of GBC-SD cells. **(B)** Comparison of collagen gel contraction of GBC-SD cells in different groups: A significant difference of gel contraction index (CI) of GBC-SD cells was observed between control group and TIMP2 or NCTD group from 1 to 4 days (*P < 0.01, *vs*. TIMP2 or NCTD group), without different CI between TIMP-2 group and NCTD group.

The collagen gel contraction test was used to determine the effect of NCTD on migration of GBC-SD cells. As shown in Figure 3, migrated potential i.e. collagen gel contraction of GBC-SD cells in control group was increased, as time prolonged. But in TIMP-2 or NCTD group with increase of the concentration, migrated potential and collagen gel contraction index (CI) of GBC-SD cells were decreased significantly, when compared with control group (all *P* < 0.01). However, no difference of GBC-SD cells’ CI was observed between TIMP-2 group and NCTD group from 1 to 4 days. It was showed that the same as TIMP-2, NCTD inhibited significantly migration of GBC-SD cells *in vitro*.

### NCTD inhibits VM-like network formation of GBC-SD cells *in vitro*

**Figure 4:**
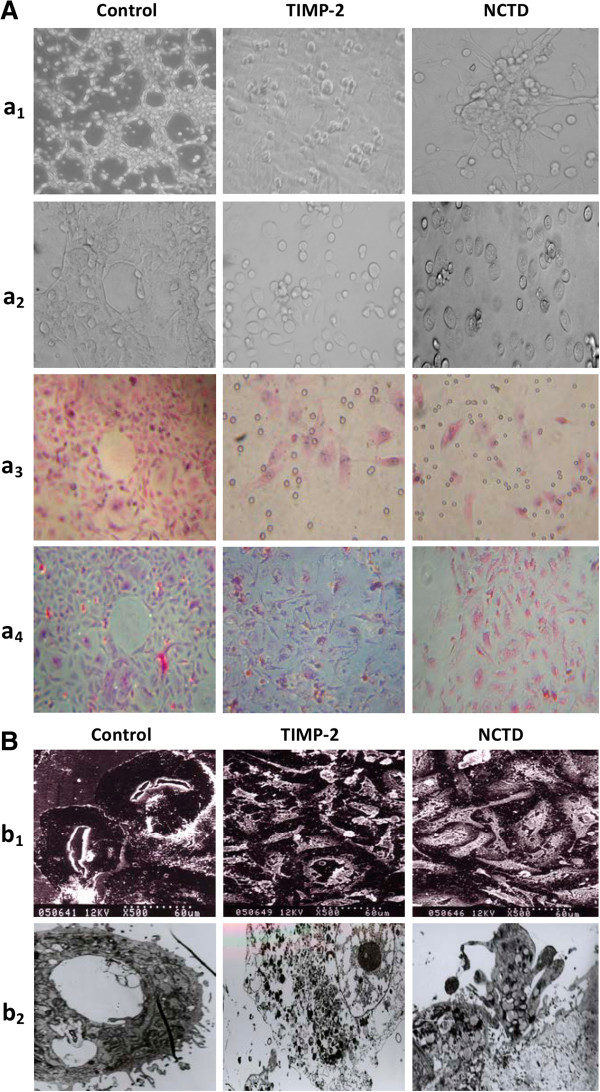
**Phase contrast microscopy and electron microscopy on 3-D cultures of GBC-SD cells *in vitro*. (A)** Phase contrast microscopy of GBC-SD cells 3-D cultured on Matrigel and rat-tail collagen I matrix (original magnification, ×200) *in vitro*. GBC-SD cells formed patterned, vasculogenic-like networks when cultured on Matrigel **(a**_**1**_**)** and rat-tail collagenImatrix (**a**_**2-3**_, H&E staining; **a**_**4**_, PAS staining without hematoxylin counterstain) for 14 days; furthermore, PAS positive, cherry-red materials found in granules and patches in the cytoplasm of GBC-SD cells appeared around the signal cell or cell clusters. But in the process of network formation, using TIMP-2 or NCTD for 2 days, GBC-SD cells lost the capacity of the above vasculogenic-like network formation, with visible cell aggregation, float, nuclear fragmentation, apoptosis and necrosis. **(B)** Vasculogenic-like network microstructures in 3-D cultures of GBC-SD cells under electron microscopies (**b**_**1**_, SEM × 500; **b**_**2**_, TEM × 1200). SEM or TEM clearly visualized channelized or hollowed vasculogenic-like networks formed GBC-SD cells ***(red arrowhead)***, with clear microvilli surrounding cluster of tumor cells; also, TEM showed some microvilli on the outside of network, clear cellular organelle structures, and cell connection with an increased electron density in density ***(yellow arrowhead)***. After using TIMP-2 or NCTD for 2 days, GBC-SD cells couldn’t grow along with collagen framework, raised and deformed, lost the capacity of the above network formation ***(blue arrowhead)***, with visible decreased microvilli, destroyed cellular organelles, Vacuolar degeneration ***(green arrowhead)***, nuclear fragmentation, and typical apoptotic bodies ***(brown arrowhead)***.

Vasculogenic-like networks formed from the 3-D cultures of GBC-SD cells *in vitro* was observed under an inverted phase-contrast light microscope and electron microscopies. As shown in Figure 4, GBC-SD cells were able to form network of hollow tubular structures when cultured on Matrigel and rat-tail collagen type I composed of the ECM gel in the absence of endothelial cells and fibroblasts (Figure 4Aa_1_-_4_). The tumor-formed networks initiated formation within 48 hr after seeding the cells onto the matrix with optimal structure formation achieved by two weeks. To address the role of the PAS positive materials in tubular networks formation and to make sure whether GBC-SD cells could secret PAS positive materials appeared around the single cell or cell clusters, 3-D cultures of GBC-SD cells were stained with PAS without hematoxylin counterstain. Microscopic analysis demonstrated that as an ingredient of the basemembrane of VM, PAS positive materials were located in granules and patches in the cell cytoplasm (Figure 4Aa_4_). SEM and TEM clearly visualized channelized or hollowed vasculogenic-like networks formed GBC-SD cells (Figure 4Bb_1_-_2_), with clear microvilli surrounding cluster of tumor cells; also, TEM showed some microvilli on the outside of network, clear cellular organelle structures, and cell connection with an increased electron density in density (Figure 4Bb_2_). In the process of vasculogenic-like structure formation, after using TIMP-2 or NCTD for 2 days, GBC-SD cells lost the capacity of the above network formation, with visible cell aggregation, float, nuclear fragmentation, apoptosis and necrosis. Furthermore, using TIMP-2 or NCTD for 48 hr after network formation, the formed vasculogenic-like structures were destructed, with visible cell aggregation, float, nuclear fragmentation and apoptosis (Figure 4Aa_1_-_4_). At the same time, SEM and TEM showed GBC-SD cells couldn’t grow along with collagen framework, raised and deformed, lost the capacity of the above network formation, with visible decreased microvilli, destroyed cellular organelles, vacuolar degeneration, nuclear fragmentation, and typical apoptotic bodies (Figure 4Bb_1_-_2_). It was thus showed that the same as TIMP-2, NCTD inhibited and destroyed forming-VM and formed-VM from 3-D cultures of GBC-SD cells *in vitro*.

### NCTD inhibits growth and VM formation of GBC-SD xenografts *in vivo*

**Figure 5:**
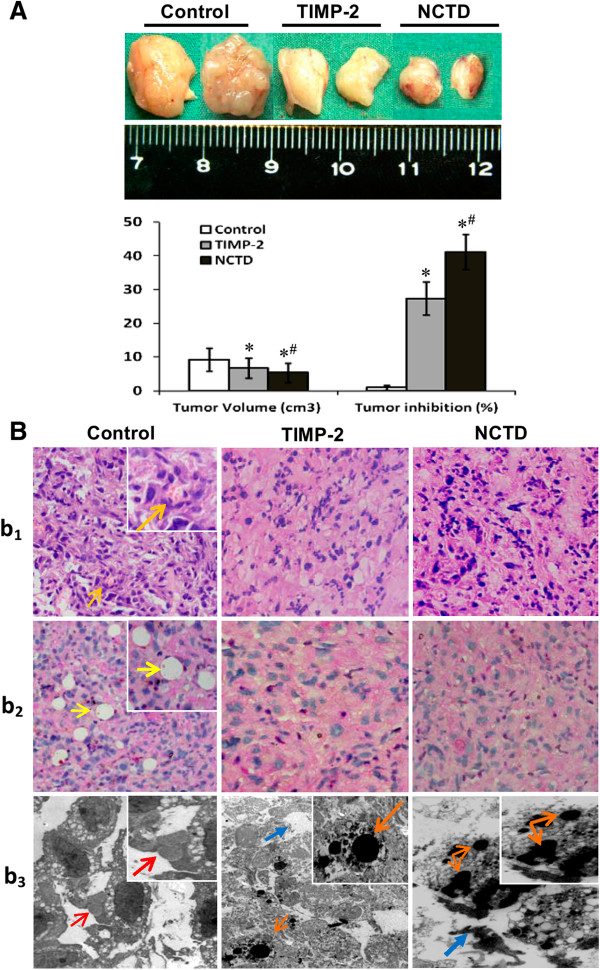
**Growth and characteristic appearance of GBC-SD xenografts *in vivo*. (A)** The size, volume and inhibition of GBC-SD xenografts of each group. *P < 0.001, *vs*. control group; ^**#**^*P* < 0.01, *vs*. TIMP-2 group. **(B)** Histomorphologic appearance of the xenografts of each group. Using H & E staining **(b**_**1**_**)** and CD_31_-PAS double stainings **(b**_**2**_**)** (original magnification, ×200), sections of the xenografts in control group showed tumor cell-lined channels containing red blood cells ***(orange arrowhead)*** without any evidence of tumor necrosis. PAS-positive substances line the channel-like structures; tumor cells form vessel-like structure with single red blood cell inside ***(yellow arrowhead)***. TEM (**b**_**3**_; original magnification, ×8000) clearly visualized several red blood cells in the centre of tumor nests in the xenografts in control group ***(red arrowhead)***. However, similar phenomenon failed to occur in the xenografts in TIMP-2 group or NCTD group, with destroyed cellular organelles, cell necrosis ***(blue arrowhead)***, nuclear pyknosis, fragmentation and apoptotic bodies ***(brown arrowhead)***.

GBC-SD xenografts appeared gradually in subcutaneous area of right axilback of nude mice from the 6^th^ day after inoculation, were in all nude mice (7/7, 100%) after 3 weeks. At the end of the experiment, the size or volume of the xenografts in NCTD or TIMP-2 group was decreased significantly in comparison with control group, with increased tumor inhibition (Figure 5A, all *P* < 0.001), and tumor inhibitory rate in NCTD group were much less than that of TIMP-2 group (*P <* 0.01).

The histological characteristics of the xenografts were observed *via* H&E staining and CD_31_-PAS double stainings under an optic microscopy and a TEM. Microscopically, the xenografts in control group showed tumor cell-lined channels containing red blood cells (Figure 5Bb_1_) without any evidence of tumor necrosis. The channel consisted of tumor cells was negative of CD_31_ and positive PAS. Tumor cells form vessel-like structure with single red blood cell inside (Figure 5Bb_2_). In the central area of tumor, the xenografts exhibited VM in the absence of ECs, central necrosis and fibrosis (Figure 5Bb_2_). Furthermore, TEM clearly showed single, double, and several red blood cells existed in the central of the tumor nests without necrosis and fibrosis in control group, and there was no vascular structure between the surrounding tumor cells and erythrocytes (Figure 5Bb_3_). However, similar phenomenon failed to occur in the xenografts in TIMP-2 or NCTD group, with destroyed cellular organelles, cell necrosis, nuclear pyknosis, fragmentation and apoptotic bodies (Figure 5Bb_3_). These findings demonstrated that VM existed in GBC-SD nude mouse xenografts and that NCTD, the same as TIMP-2, inhibited the VM formation of GBC-SD nude mouse xenografts *in vivo*.

### NCTD affects VM’ hemodynamic of GBC-SD xenografts *in vivo*

**Figure 6:**
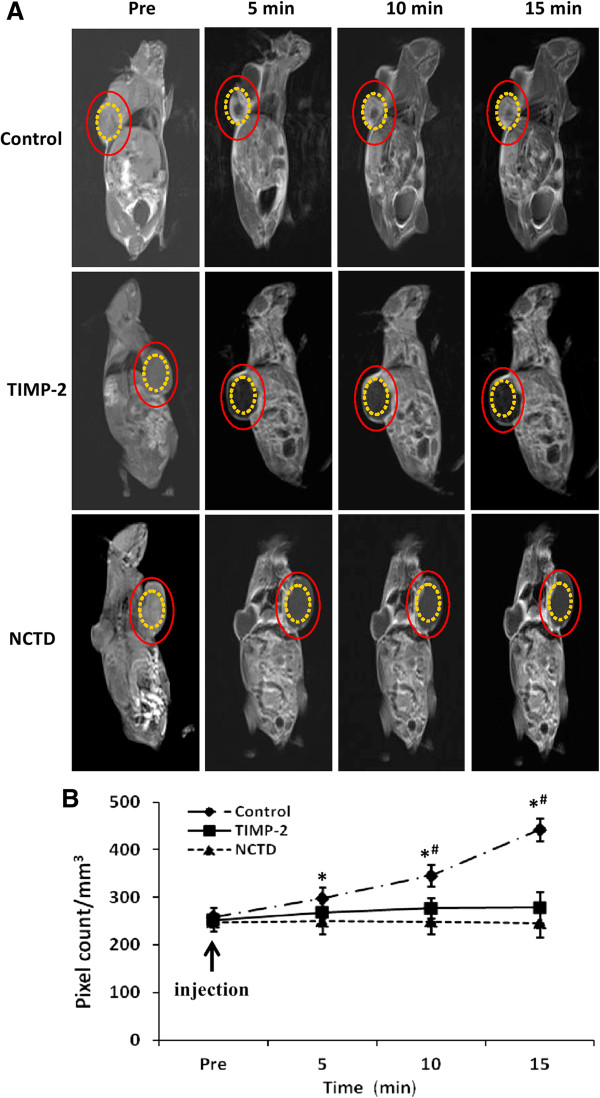
**Dynamic micro-MRA and hemodynamic of GBC-CD xenografts *in vivo*. (A)** The images were acquired before the injection (pre), 5, 10, and 15 min after injection of the contrast agents (HAS-Gd-DTPA). The tumor center area ***(yellow circle)*** in control group exhibited a signal that gradually increased multiple spots in intensity (which is consistent with the intensity observed in tumor marginal area between the ***(red circle*** and the ***yellow circle)***. However, the center region ***(yellow circle)*** of the xenografts in NCTD or TIMP-2 group exhibited a decreased signal or a lack of signal change in intensity. **(B)** Hemodynamic changes of the xenografts’ VM of each group. All data are expressed as means ± SD. *P < 0.001, *vs.* Pre injection in control group; ^**#**^*P* = 0.0000, *vs.* NCTD or TIMP-2 group. But, no difference on signal intensity (pixel count/mm^3^) was observed between NCTD group and TIMP-2 group.

Two-mm-interval horizontal scanning of GBC-SD xenografts of each group were conducted to compare tumor signal intensities of the xenograft mice by dynamic Micro-MRA with an intravascular macromolecular MRI contrast agent named HAS-Gd-DTPA. We found that the tumor center of GBC-SD xenografts in control group exhibited a signal that gradually increased multiple high-intensity spots, i.e., higher occurrence of VM observed in tumor center of the xenografts with gradual increased high-intensity MRI signal, a result consistent with pathological VM (Figure 6AB, Table 2). However, the center region of the xenografts in NCTD or TIMP-2 group exhibited a low intensity signal or a lack of signal change in intensity, a result consistent with central ischemic disappearance of nuclei, and apoptosis; and no difference on signal intensity (pixel count/mm^3^) was observed between NCTD group and TIMP-2 group (Figure 6AB). It deduced that NCTD inhibits the xenografts’ growth, induces the ischemic necrosis of the xenografts by suppressing hemodynamic and VM of the xenografts.

**Table 2:** Relationship between VM and MRI image in mice with GBC-SD xenografts

**MRI signal intensity in tumor center**	**MRI (n)**	**VM (n)**		***P***
		**(+)**	**(-)**	
Gradual increase	5	5	0	0.0000
No increase	13	0	13	

### NCTD downregutates expression of VM signaling-related markers PI3-K, MMP-2, MT1-MMP and Ln-5γ2 *in vitro* and *in vivo*

**Figure 7:**
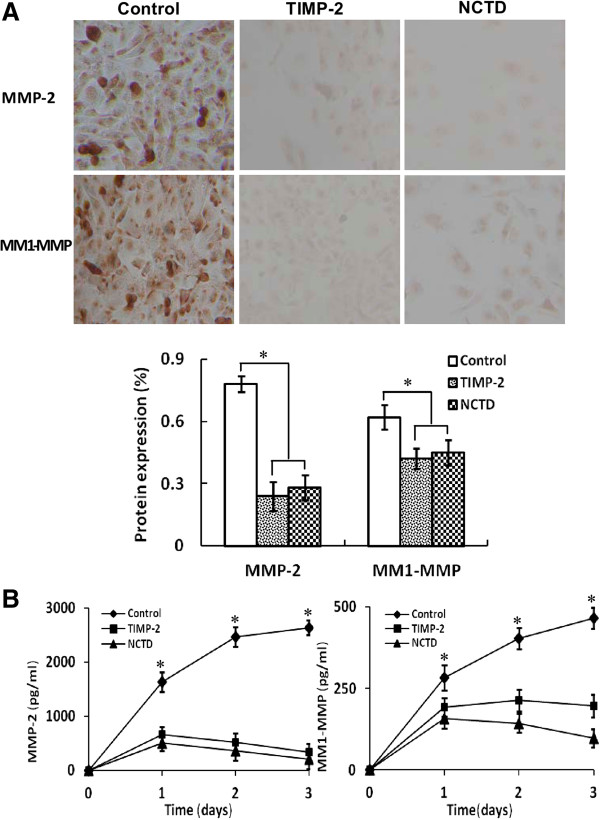
**Expression of MMP-2, MT1-MMP proteins from sections [(A), SABC method, original magnification, ×200)] and supernates [(B), ELISA] of the 3-D cultures of GBC-SD cells *in vitro*. (A)** The positive expression site of MMP-2 and MT1-MMP proteins presented yellow-brown reactant was in cytoplast. Expression of MMP-2 and MT1-MMP proteins in TIMP-2 group and NCTD group was significantly lower than that of control group (all *P < 0.001). **(B)** Expression of MMP-2 and MT1-MMP proteins from supernates of 3-D culture samples in control group increased significantly as time prolonged, when compared with TIMP-2 group or NCTD group (all *P < 0.001).

**Figure 8:**
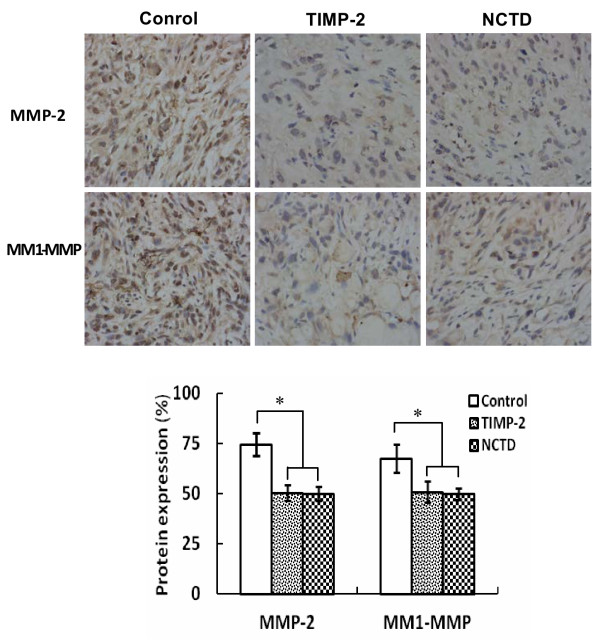
**Expression of MMP-2, MT1-MMP proteins from sections of GBC-CD xenografts *in vivo *(SABC method, original magnification × 200).** Overexpression of MMP-2 and MT1-MMP proteins in control group was all observed *in vivo*. Expression of MMP-2 and MT1-MMP proteins in TIMP-2 group and NCTD group was significantly decreased (*P < 0.001, *vs.* control group).

**Figure 9:**
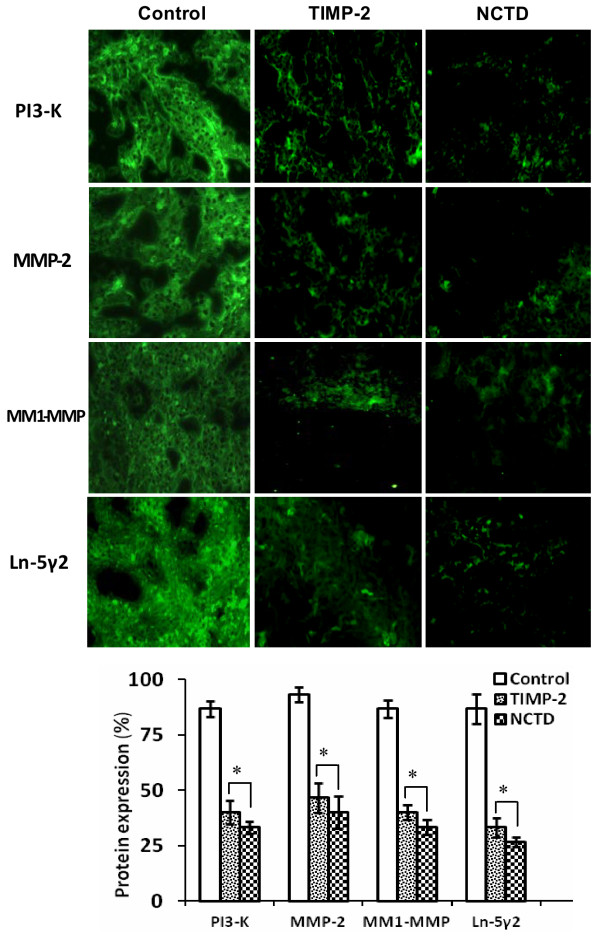
**Expression of VM signal-related proteins PI3-K, MMP-2, MM1-MMP and Ln-5γ2 of GBC-CD xenografts *in vivo *(indirect immunofluorescence method, original magnification, ×400).** The positive expression site of these proteins presented bright yellow-green fluorescent staining reactant was all in cytoplast. Expression of PI3-K, MMP-2, MM1-MMP and Ln-5γ2 proteins in control group was all upregulated markedly. However, expression of these proteins in TIMP-2 group and NCTD group was significantly downregulated (*P < 0.001, *vs.* control group). But, no difference on expression of these proteins was observed between NCTD group and TIMP-2 group.

**Figure 10:**
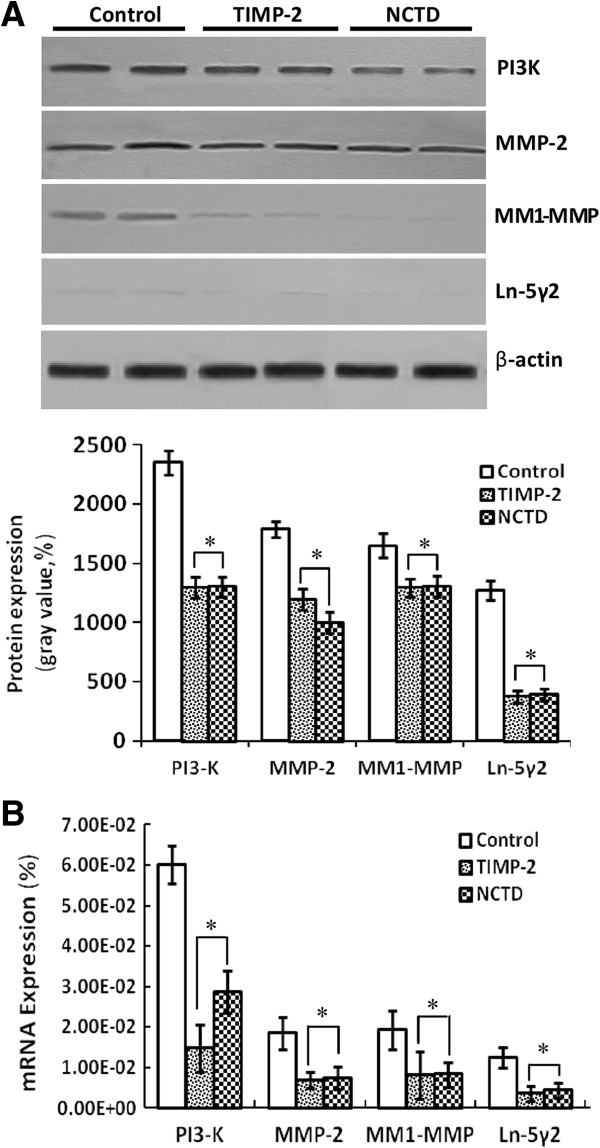
**Expression of VM signal-related proteins/mRNAs PI3-K, MMP-2, MM1-MMP and Ln-5γ2 of GBC-CD xenografts *in vivo*. (A)** Western blotting: Overexpression of PI3-K, MMP-2, MM1-MMP and Ln-5γ2 proteins of GBC-SD xenografts in control group was observed; but expression of these proteins in TIMP-2 or NCTD group was significantly decreased (*P < 0.001, *vs*. control group). **(B)** RT-PCR: Expression of PI3-K, MMP-2, MM1-MMP and Ln-5γ2 mRNAs of GBC-SD xenografts in control group was increased significantly when compared with TIMP-2 and NCTD groups (*P < 0.01).

To investigate the underlying mechanisms of NCTD effects on tumor growth and VM of human GBCs *in vitro* and *in vivo*, in this study we explored the regulation effect of NCTD on the PI3-K/MMPs/Ln-5γ2 signaling pathway i.e., expression of VM signaling-related markers PI3-K, MMP-2, MT1-MMP and Ln-5γ2 *in vitro* and *in vivo*. Expression of MMP-2 and MT1-MMP proteins from sections and supernates of 3-D culture samples of GBC-SD cells *in vitro* were examined by SABC and ELISA, and expression of PI3-K, MMP-2, MT1-MMP and Ln-5γ2 at protein and mRNA levels from sections of GBC-SD xenografts *in vivo* were determined by SABC, indirect immunofluorescence, Western blotting and RT-PCR. We found that in sections of 3-D culture samples of GBC-SD cells *in vitro*, the positive expression site of MMP-2 and MT1-MMP proteins presented yellow-brown reactant in the cytoplasm; overexpression of MMP-2 and MT1-MMP proteins in control group was observed, expression of MMP-2 and MT1-MMP proteins in TIMP-2 or NCTD group was significantly lower than that of control group (Figure 7A; all *P < 0.001); expression of MMP-2 and MT1-MMP proteins from supernates of 3-D culture samples *in vitro* in control group increased significantly as time prolonged, when compared with TIMP-2 or NCTD group (Figure 7B; all *P < 0.001). And, overexpression of MMP-2, MT1-MMP proteins from sections of GBC-SD xenografts in control group was all observed *in vivo*; expression of MMP-2 and MT1-MMP proteins of the *in vivo* xenografts in TIMP-2 or NCTD group was significantly lower than that of control group (Figure 8; all *P < 0.001). Furthermore, it was *in vivo* showed that not only expression (bright yellow-green fluorescent staining reactant in cytoplast, or Western gray value) of PI3-K, MMP-2, MM1-MMP and Ln-5γ2 proteins in control group was all upregulated markedly, with significantly downregulated expression of these VM signaling-related proteins in TIMP-2 or NCTD group (Figures 9 and 10A; all *P < 0.001), but also, expression of PI3-K, MMP-2, MM1-MMP and Ln-5γ2 mRNAs of GBC-SD xenografts in TIMP-2 or NCTD group was decreased significantly when compared with control group (Figure 10B; all *P < 0.01); and no difference on expression of these VM signaling-related proteins/mRNAs was observed between NCTD group and TIMP-2 group. We previously reported that highly aggressive GBC-SD cells overexpressed MMP-2, MT1-MMP, PI3-K and Ln-5γ2 formed *in vitro* and *in vivo* VM networks through the activation of the PI3-K/MMPs/Ln-5γ2 signaling pathway, the PI3-K/MMPs/Ln-5γ2 signaling pathway contributed to vasculogenic mimicry of human gallbladder carcinoma GBC-SD cells *in vitro and in vivo*, and TIMP-2 effectively inhibit expression of these VM signaling-related markers, thus inhibiting VM of GBC-SD cells *in vitro and in vivo*[22]. The results in this study showed that NCTD downregulated expression of VM signaling-related markers PI3-K, MMP-2, MT1-MMP and Ln-5γ2 *in vitro* and *in vivo*, and similarly to TIMP-2, inhibited the VM formation of GBC-SD cells *in vitro* and GBC-SD nude mouce xenografts *in vivo*. These findings firstly demonstrated that NCTD inhibits tumor growth and VM of human GBCs by suppression of the PI3-K/MMPs/Ln-5γ2 signaling pathway *in vitro* and *in vivo*.

## Discussion

Because of highly aggressive characteristic, disappointing surgical resection and chemo-radiotherapies, and poor prognosis of the patients with GBC, novel adjuvant therapies or anticancer agents are clearly needed to treat this disease [1-12]. Considering an effective tumor microcirculation consists of vasculogenesis, angiogenesis and VM, many researchers are currently seeking to develop new angiogenic and VM inhibitors from cleaved proteins, monoclonal antibodies, synthesized small molecules and natural products [34-40]. Some angiogenic inhibitors such as bevacizumab (Avastin, a VEGF inhibitor), sorafenib, erlotinib, sunitinib, angiostatin, endostatin, thrombospondin-1, celastrol, TNP-470, and SU-5416 have been reported to exhibit antitumor and antiangiogenic activities [36,41-46]. However, recent studies have suggested that the benefits of antiangiogenic therapy have far been rather modest, also, sole blockage of angiogenesis may not be effective [25,47,48]. VM is believed as a special blood passage conspicuously different from angiogenesis in some highly aggressive malignant tumors, it should be so considered to develop new antivascular therapeutic agents that target both angiogenesis and VM, in especial, anti-VM therapy for VM when in antitumor treatment of some highly aggressive tumors with VM. McNamara MG et al have suggested that the future therapeutic spectrum for GBC will likely encompass novel combinations of targeted therapies with cytostatics in scientifically and molecularly directed schedules, thus permitting fewer mechanisms of escape for tumor cells [13].

NCTD, a demethylated and low-cytotoxic derivative of cantharidin, not only inhibits the proliferation and growth of a variety of human tumor cells and is used clinically to treat some human cancers because of its anticancer activity, fewer side effects and leukocytosis [26-31], but has multiple antitumor activities against GBCs *in vitro* and *in vivo*[32-34]. In this study, we further investigated the anti-VM activity of NCTD as a VM inhibitor for human GBCs. The results have shown that GBC-SD cells were able to form vasculogenic-like network structures when cultured on 3-D matrices and seeded into the axilback of nu/nu mice, and then facilitated growth of GBC-SD cells or xenografts, which were concordant with our previous reports [22,24]; that NCTD inhibited significantly proliferation, invasion, migration, vasculogenic-like network formation of GBC-SD cells *in vitro*, and suppressed VM formation and VM hemodynamic of GBC-SD xenografts *in vivo*, then inhibiting tumor xenografts’ growth. Thus, we concluded that NCTD may be a potential anti-VM agent for human GBCs.

Molecular events underlying VM displayed by highly aggressive malignant tumor cells such as aggressive human GBCs remain poorly understood. Therefore, understanding the key molecular mechanisms that regulate VM in human GBCs would be an important event and provide potential targets for new therapies of GBCs. Recently, experimental evidences have shown the importance of several key molecules or signaling pathways in the formation of vasculogenic-like networks by aggressive malignant tumor cells, including PI3K, MMPs, Ln­5γ2 chain [49-52], etc. PI3-K/MMPs/Ln-5γ2 signaling pathway is a key pathway which regulated VM formation of aggressive malignant tumor cells. PI3­K is a smaller lipid kinase. Its main activity product PI(3,4,5) ­P3 acts as a binding site for many intracellular proteins. PI3­K signaling plays an integral role in many normal cellular processes, including survival, proliferation, differentiation, metabolism and motility, in a variety of cell types [53]. MMPs, divided into soluble MMPs and MT-MMP, are a broad family of zinc-biding endopepeidases that participate in the ECM degradation that accompanies cancer cell invasion, metastasis, and angiogenesis [54-56]. Recent studies have indicated that MMP-2 and MT1-MMP expression was significantly related to VM formation in melanoma and ovarian carcinoma cells in 3-D culture [49,52]. The Ln-5γ2 chain, MMP-2, and MT1-MMP act cooperatively and required for highly aggressive melanoma tumor cells to engage in VM when cultured on a 3-D ECM [50]. The Ln-5γ2 chain in the ECM is able to romote VM formation [50,51]. As an important adjustor, PI3-K directly affects the cooperative interactions of MT1-MMP and MMP-2 activity in highly aggressive melanoma cells, and regulates MT1-MMP activity which promotes the conversion of pro-MMP into its active conformation through an interaction with TIMP-2. Both enzymatically active MT1-MMP and MMP-2 may then promote the cleavage of Ln-5γ^2^ chains into promigratory γ^2^ and γ^2x^ fragments. The deposition of these fragments into tumor extracellular milieu may result in increased migration, invasion and VM formation [50,51]. Special inhibitors of PI3­K may impair VM formation and decrease MT1-MMP and MMP-2 activity; inhibition of PI3­K blocked the cleavage of Ln-5γ2 chain, resulting in decreased levels of the γ2, and γ2× pro­migratory fragments [49]. We reported that highly aggressive GBC-SD cells overexpressed MMP-2, MT1-MMP, PI3-K and Ln-5γ2, formed VM in human GBCs through the activation of the PI3K/MMPs/Ln-5γ2 signaling pathway *in vitro* and *in vivo*; and the PI3-K/MMPs/Ln-5γ2 signaling pathway contributed to VM of human GBC cells *in vitro and in vivo*[23,24]. So, the PI3­K/MMPs/Ln-5γ2 signaling pathway may represent predominant targets for anti-VM of tumors and cancer therapy. In this study, we explored the regulation effect of NCTD on the PI3-K/MMPs/Ln-5γ2 signaling pathway i.e., expression of VM signaling-related markers PI3-K, MMP-2, MT1-MMP and Ln-5γ2. The results have showed that NCTD downregulated expression of these VM signaling-related markers *in vitro* and *in vivo*; thus inhibited the VM formation of GBC-SD cells *in vitro* and GBC-SD nude mouse xenografts *in vivo*. These findings demonstrated that NCTD inhibits tumor growth and VM of human GBCs by suppression of the PI3-K/MMPs/ Ln-5γ2 signaling pathway.

TIMP-2 is a 21-kDa protein which selectively forms a complex with the latent proenzyme form of the 72-kDa type IV collagenase, thereby inhibits the type IV collagenolytic activity and the gelatinolytic activity, and abolishes the hydrolytic activity of all members of the metalloproteinase family [24]. TIMP-2 is a potent inhibitor of cancer cell invasion through reconstituted ECM [57]. Addition of endogenous inhibitor TIMP-2 or antibodies to 72-kDa type IV collagenase or specific antiserum against the 72-kDa type IV collagenase achieved the alteration of the type IV collagenase-inhibitor balance, then inhibited HT-1080 cell invasion [57]. A significantly higher concentration of TIMP-2 may effectively inhibit all of the proteolytic activities associated with MMP-2 and/or MT1-MMP. The inhibition of either MMP-2 or MT1-MMP activity with antibodies is sufficient to prevent formation of vasculogenic-like patterned networks [50]. We reported that recombinant TIMP-2 retarded patterned VM formation in 3-D matrices of GBC-SD and xenografts within 2 weeks of seeding and injecting, and downregulated expression of MMP-2, MT1-MMP, PI3-K and Ln-5γ2 proteins/mRNAs *in vitro and in vivo*, whereas all of untreated GBC-SD cells and xenografts formed vasculogenic-like patterned networks, upregulated expression of these VM signaling-related proteins/mRNAs; so believed that TIMP-2 inhibited VM formation of GBC-SD cells *in vitro and in vivo* through suppression of the PI3-K/MMPs/Ln-5γ2 signaling pathway [24]. In this study, we designed TIMP-2 as an experimental control group, to investigate comparatively the inhibitory effect of NCTD on VM in GBCs and its mechanism. The results showed that NCTD, similarly to TIMP-2, not only inhibited the VM formation of GBC-SD cells and xenografts, but also downregulated expression of PI3-K, MMP-2, MT1-MMP and Ln-5γ2 *in vitro* and *in vivo*; therefore, served as a disproof that NCTD inhibits tumor growth and VM of human GBCs by suppression of the PI3-K/MMPs/ Ln-5γ2 signaling pathway *in vitro* and *in vivo*.

## Conclusions

Collectively, NCTD inhibits tumor growth and VM of human GBCs by suppression of the PI3-K/MMPs/Ln-5γ2 signaling pathway. NCTD could serve as a potential anti-VM agent for human GBCs. It should be considered to use this VM inhibitor when in antitumor treatment of some highly aggressive tumors with VM.

## Competing interests

The authors declare that they have no competing interests.

## Authors’ contributions

ZJT, SW, ZWZ, and FYZ designed the research, analyzed the data and wrote the manuscript. SW carried out *in vitro* experiments and 3-D culture of GBC-SD cells. ZWZ, GCY and LZY carried out *in vivo* experiments of GBC-SD nude mouse xenografts. SW and ZWZ were responsible for the existence of VM *via* immunohistochemistry staining, SEM or TEM and micro-MRA technology, respectively. GCY and LZY were responsible for the detection of VM signaling-related markers. FYZ is the guarantor. All authors have read and approved the final manuscript.

## Pre-publication history

The pre-publication history for this paper can be accessed here:

http://www.biomedcentral.com/1471-2407/14/193/prepub
